# Modulating Activity in the Dorsolateral Prefrontal Cortex Changes Punishment in the 3-Player Prisoner’s Dilemma: A Transcranial Direct Current Stimulation Study

**DOI:** 10.3389/fnins.2019.01160

**Published:** 2019-10-25

**Authors:** Shu Chen, Jinchuan Shi, Xiaolan Yang, Hang Ye, Jun Luo

**Affiliations:** ^1^College of Economics, Interdisciplinary Center for Social Sciences, Zhejiang University, Hangzhou, China; ^2^Institute for Applied Microeconomics, University of Bonn, Bonn, Germany; ^3^Academy of Financial Research, Zhejiang University, Hangzhou, China; ^4^School of Business and Management, Shanghai International Studies University, Shanghai, China; ^5^Center for Economic Behavior and Decision-Making, School of Economics, Zhejiang University of Finance and Economics, Hangzhou, China

**Keywords:** punishment, cooperation, 3-player prisoner’s dilemma, dorsolateral prefrontal cortex, transcranial direct current stimulation

## Abstract

Altruistic punishment of social norm violations plays a crucial role in maintaining widespread cooperation in human societies, and punitive behavior has been suggested to be related to the activity level of the dorsolateral prefrontal cortex (DLPFC). This study used unilateral and bilateral transcranial direct current stimulation (tDCS) to investigate how modulating the activity of the DLPFC affects cooperation and punishment in a 3-player prisoner’s dilemma. We found that none of the unilateral stimulations changed the participants’ cooperation behaviors, while left anodal/right cathodal stimulation increased the participants’ cooperation. For punitive behavior, we found that all unilateral stimulations (i.e., right anodal, right cathodal, left anodal, left cathodal) and bilateral stimulations (i.e., right anodal/left cathodal, left anodal/right cathodal) significantly decreased the punishment imposed by the cooperators toward the defectors. In addition, right anodal stimulation significantly decreased the participant’s third-party punishment (TPP) imposed by the cooperators toward the defectors. The other three unilateral stimulations also significantly decreased the participant’s TPP imposed by the cooperators toward the defectors, but only when the punishment was revealed to the punished person. Our findings indicate that the mechanisms of selfishness and negative emotions suggested by previous studies probably interact with different stimulations: for anodal stimulations, the mechanism of negative emotions may overwhelm the mechanism of selfishness, while for cathodal stimulations, the mechanism of selfishness may be more dominant than the mechanism of negative emotions.

## Introduction

Altruistic punishment of social norm violations plays a crucial role in maintaining widespread cooperation in human societies, even though imposing punishment has a cost to the punishers (Gächter and Fehr, 1999; Fehr and Gachter, 2000; Fehr and Gächter, 2002; Fehr and Fischbacher, 2004). A common method of investigating norm violation and punishment is by using interactive economic games (Camerer, 2003a, b; Fehr and Camerer, 2007; Sanfey, 2007), such as the ultimatum game (Güth et al., 1982; see Gabay et al., 2014 for a meta-analysis) and the prisoner’s dilemma (Dickinson et al., 2015). Behavioral findings indicate that norm violations lead to punishments by other individuals at monetary expense in these games. Neuroscientific studies have identified an important role of the dorsolateral prefrontal cortex (DLPFC) in the neuropsychological networks that mediate altruistic punishment (Fehr and Camerer, 2007; Harlé and Sanfey, 2012; Feng et al., 2016; Wang et al., 2017). It is well known that the activity of the DLPFC is associated with implementing self-control and cognition processes (Miller and Cohen, 2001; Knoch et al., 2006; Cohen and Lieberman, 2010), but these processes can affect the decision-making of altruistic punishment in various ways, resulting in sometimes seemingly contradictory results.

One way that DLPFC mediates the decision-making of altruistic punishment is by affecting people’s self-control toward selfishness (Knoch et al., 2006, 2007). Specifically, the activation of the DLPFC enables people to better control their selfishness, thus leading to more selfless behaviors. In the context of altruistic punishment, this means that people will be more willing to punish norm violators at their own expense if they have a higher level of activation in the DLPFC. In contrast, a lower level of activation in the DLPFC will result in issuing lower altruistic punishment. For simplicity, we denote this as the mechanism of selfishness. Some non-invasive brain stimulation (NIBS; i.e., repetitive transcranial magnetic stimulation, rTMS, or transcranial direct current stimulation, tDCS) studies have found that inhibiting the activity of the DLPFC decreases the rate of rejection toward unfair offers in the ultimatum game, which is regarded as a typical kind of altruistic punishment (Knoch et al., 2006, 2007; Baumgartner et al., 2011). Buckholtz et al. (2015) also found that inhibiting the activity of the DLPFC decreases altruistic punishment in the context of crimes. Moreover, this relationship between the activity in the DLPFC and altruistic punishment is suggested by a neuroscientific study that measured the participants’ resting-state electroencephalography (EEG) activity (Knoch et al., 2010) before they imposed punishments. A positive relationship was revealed between the resting-state alpha activity of the right DLPFC and the likelihood of an altruistic punishment.

The other way that the DLPFC mediates the decision-making of altruistic punishment is by affecting people’s self-control toward negative emotions (Sanfey et al., 2003; Wu and Zhou, 2012; Luo et al., 2013; Yin et al., 2019). In detail, the activation of the DLPFC enables people to better control their negative emotions toward norm violators, thus making them not as willing to punish these norm violators. As a result, people will impose lower altruistic punishment if they have a higher level of activation in DLPFC, and a lower level of activation in DLPFC will result in imposing higher altruistic punishment, which is exactly opposite to the selfishness mechanism above (we denote it as the mechanism of negative emotions). This negative emotion mechanism is also supported by brain stimulation studies. For example, Brüne et al. (2012) used TMS and the dictator game with third-party punishment (TPP) and found that inhibiting the activity of the right DLFPC by TMS increased the participants’ rate of punishment. In addition, a functional magnetic resonance imaging (fMRI) study observing the activity level of the DLPFC when the participants were making punitive decisions found that participants who did not punish displayed a higher activity level in the right DLPFC and a lower activity level in the left DLPFC (Brüne et al., 2013).

This study is an effort to reconcile the above seemingly contradictory results about the causal relationship between the activity of the DLPFC and altruistic punishment, in which we used unilateral and bilateral tDCS to investigate how modulating the activity of the DLPFC corresponds to altruistic punishment in a 3-player prisoner’s dilemma. Our contributions are as follows. First, previous studies applied different stimulation methods, different experimental tasks, and even different kinds of punishment, making it difficult to compare them with each other. In this study, we investigated the effect of enhancing or inhibiting the activity of each unilateral DLPFC for a comparable analysis to explore how the mechanisms of selfishness and negative emotions function in the decision-making of altruistic punishment. Second, previous studies usually regarded the rejection of unfair offers in the ultimatum game as altruistic punishment but seldom pay attention to altruistic punishment in the prisoner’s dilemma, which is another typical game studied in experimental economics. However, we would like to know whether the DLPFC also play an important role in the altruistic punishment from the prisoner’s dilemma and whether the mechanisms of selfishness and negative emotions still apply to this kind of altruistic punishment. Third, previous studies do not consider the characteristics of the imposer and the receiver of the punishment. In fact, behavioral studies have shown that the characteristics of the punished person plays the greatest role in the punishment (Falk et al., 2005). In our study, we distinguished punishment into four types according to the characteristics of the imposer and the receiver of the punishment and investigated how different types of stimulation affect different types of punishment. Last, previous studies did not compare second-party punishment (SPP) and TPP at the same time, nor did they compare the effect of changing the balance of the bilateral DLPFC but instead modulated solely the activity in each unilateral DLPFC. This study also investigates these aspects to see whether the DLPFC functions differently in the decision-making of SPP and TPP, as well as whether changing the balance of the bilateral DLPFC and modulating solely the activity in each unilateral DLPFC have different results toward altruistic punishment.

For each type of punishment investigated in this study, our hypotheses are as follows. If we observed that enhancing the activity in the DLPFC increased altruistic punishment and that inhibiting the activity in the DLPFC decreased altruistic punishment, then the mechanism of selfishness probably prevails over the mechanism of negative emotions in this type of punishment. If we observed the opposite, i.e., that enhancing the activity in the DLPFC decreased altruistic punishment and inhibiting the activity in the DLPFC increased altruistic punishment, then it is likely that the mechanism of negative emotions is more dominant compared to the mechanism of selfishness in this type of punishment. However, if we do not observe opposite effects when enhancing and inhibiting the activity in the DLPFC, then the two mechanisms of selfishness and negative emotions probably interact with different stimulations.

## Materials and Methods

### Participants

We recruited 162 participants overall to participant in our experiment. All the participants are students from the universities in the Xiasha University Town in Hangzhou (54 males, 108 females; mean age 20.78 years, 95.7% between 18 and 24 years).^1^ The participants were right-handed and declared no history of psychiatric illness or neurological disorders with no experience of tDCS or the tasks in the experiment. Before participating in the experiment, the participants were required to provide written informed consent approved by the Zhejiang University Ethics Committee. The experiment was implemented in the Center for Economic Behavior and Decision-making of Zhejiang University of Finance and Economics with duration of approximately 1.5 h. The participants received an average payment of 60.90 RMB yuan (approximately 8.85 dollars).

There were two kinds of stimulation treatments: the unilateral DLPFC stimulation treatment (including right anodal, right cathodal, left anodal, left cathodal, and unilateral sham), and the bilateral DLPFC stimulation treatment (including right anodal/left cathodal, left anodal/right cathodal, and bilateral sham). Each participant was randomly assigned to one of the stimulation modes, and we balanced the gender among different stimulation modes. The information on stimulation assignment is given in Table 1.

**TABLE 1:** Gender composition of the stimulation modes.

	**Unilateral DLPFC stimulation**					**Bilateral DLPFC stimulation**		
	**R+**	**R−**	**L+**	**L−**	**U-Sham**	**R+L−**	**L+R−**	**B-Sham**
Male	6	6	6	6	6	8	8	8
Female	12	12	12	12	12	16	16	16

### Transcranial Direct Current Stimulation

Transcranial direct current stimulation was delivered by a battery-driven stimulator (multichannel non-invasive wireless tDCS neurostimulator, Starlab, Barcelona, Spain) via two saline-soaked surface sponge electrodes (35 cm^2^) fixed on the scalp of the participant with a rubber belt. The current had a constant intensity of 1.5 mA, delivered for 20 min with 30 s of ramping up and down. This montage would induce cortical excitability changes in the target area without causing any physiological damage to the participants. The anodal electrode would enhance cortical excitability, while the cathodal electrode would inhibit it (Nitsche and Paulus, 2000). For the sham stimulation, the current was delivered only for 30 s once it reached 1.5 mA. However, the participants treated this as the regular process of the stimulation and were unaware of their stimulation types according to the questionnaire completed after the experiment. This method of sham stimulation has also been shown to be reliable in previous research (Gandiga et al., 2006).

**FIGURE 1:**
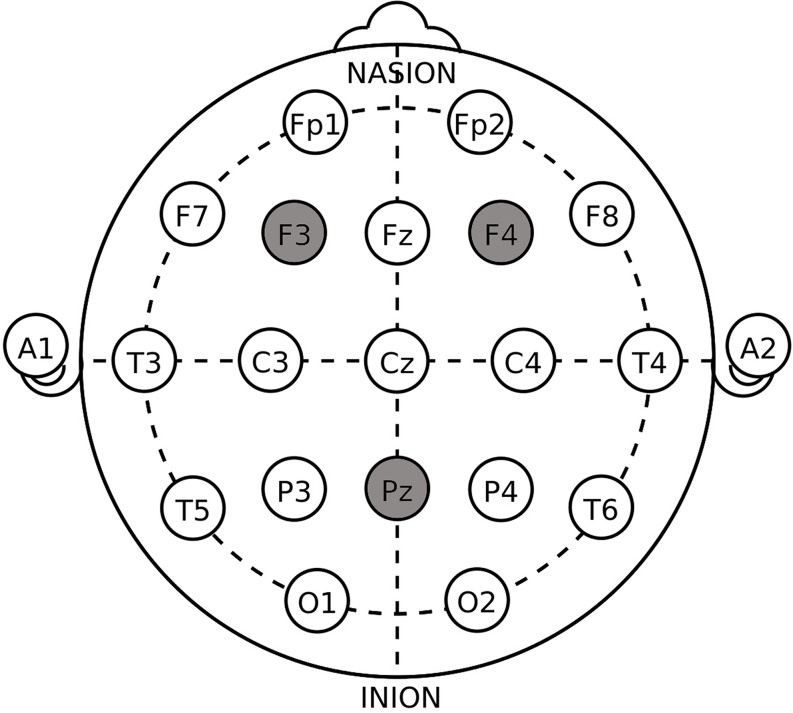
Target areas of unilateral stimulations (F_3_/F_4_ and P_*Z*_) and bilateral stimulations (F_3_ and F_4_) according to the International 10–20 System.

The target areas were localized according to the International 10–20 System (Figure 1). For the unilateral DLPFC stimulations, the anodal or the cathodal electrode was placed over the right F4 or the left F3 according to the stimulation modes whose names are self-explanatory, while the return electrode was placed over Pz. The position of Pz was chosen to construct the current circuit together with the DLPFC because of the reasonable spatial and functional distance from the parietal cortex to our target region, which decreased the possibility of stimulation interaction or task interference (Cohen et al., 2000; Simon et al., 2002; Wagner et al., 2005). The placement of the electrodes in unilateral sham was randomly selected out of the four placements above. For the bilateral DLPFC stimulations, the anodal (cathodal) electrode was placed over the right F4, and the cathodal (anodal) electrode was placed over the left F3, in the right anodal/left cathodal (left anodal/right cathodal) treatment. The placement of the electrodes in bilateral sham also randomly applied one of the two bilateral placements. These stimulation montages were suggested effective in modulating the activity of unilateral or bilateral DLPFC in previous literature (Fecteau et al., 2007a, b; Merzagora et al., 2010).

### Experiment Design

#### 3-Player Prisoner’s Dilemma

The 3-player prisoner’s dilemma is modified from Falk et al. (2005), in which three players are grouped together to decide simultaneously whether to choose Strategy A (represents cooperate) or Strategy B (represents defect). The profit of Player *i* from the choice is represented by the following formula:

$$P_{i}=20+2\times D_{i}-4\times \sum_{j=1,j\neq i}^{3}D_{j}$$

*P*_*i*_ is the profit of player *i* in the game. *D*_*i*_ = 0 if Player *i* chooses Strategy A and *D*_*i*_ = 1 if Player *i* chooses Strategy B. In this case, every defection (i.e., choosing Strategy B) will lead to two tokens’ benefit for the defector and four tokens’ harm for each of the other two group members compared to the situation in which every member cooperates (i.e., choosing Strategy A). Table 2 shows the profit matrix of the three players denoted as *i*, *j*, and *k* in a group. For each player, the optimal choice is defection given other players’ choices, while the total benefit of the group is maximized when everyone chooses cooperation. In other words, the benefits of the individuals and the group are at odds, which is the crucial characteristic of prisoner’s dilemma.

**TABLE 2:** Profit matrix of the 3-player prisoner’s dilemma.

	***D*_*j*_ = *D*_*k*_ = 0**	***D*_*j*_ = 1,*D*_*k*_ = 0**	***D*_*j*_ = *D*_*k*_ = 1**
*D*_*i*_ = 0	20, 20, 20	16, 22, 16	12, 18, 18
*D*_*i*_ = 1	22, 16, 16	18, 18, 12	14, 14, 14

*The three players are denoted as *i*, *j*, and *k*. The three numbers in each grid refer to *P*_*i*_, *P*_*j*_, and *P*_*k*_ successively.*

After the players make their decisions of choosing Strategy A or Strategy B, the computer will randomly decide the punishment type in the following punitive process. If the computer chooses SPP, the choices of the players are revealed anonymously to their group members. Each player is asked how much he/she wants to punish each of the other two players in the same group. After the punitive decision, one of the players in the group will be randomly chosen as the punisher, and one of the remaining two players will be randomly chosen to be punished. If the computer chooses TPP, then the strategies of the players are revealed anonymously to the players in another group. Specifically, all the groups in a session are randomly sorted, and the choices of the first group were shown to the players in the second group, the choices of the second group were shown to the third group, and so forth. The choices of the last group were shown to the first group. Each player is asked how much he/she wants to punish each of the players in the previous group. After the punitive decision, one of the players in each group will be randomly chosen as the punisher to punish the player in the next group and one of the remaining two players will be randomly chosen to be punished by the punisher of the previous group.

Although each player is asked to make the punitive decision, only the punishment from the selected punisher on the selected published player will be implemented. Every token that the punisher spends will deduct three tokens from the punished player. The amount of tokens the punisher spends can be any integer between 0 and 8 (including 0 and 8). Moreover, the punitive decision includes decisions under two circumstances: if the amount of punishment is revealed to the punished player at the end of the experiment (denoted as revealed condition); and if the punished player will never know how many tokens he or she has been punished (denoted as naive condition). This montage is used to investigate whether the punishment is known by the punished person plays a role in punitive decisions, and the computer will randomly choose a condition from the two conditions to implement the punishment. The payoff of the task is the profit of the 3-player prisoner’s dilemma minus the punishment cost or the punishment received.

#### Inequality Choice Menus

The inequality choice menus that we used are from Yang et al. (2016), which aims to test the participants’ levels of inequality aversion (including disadvantageous inequality aversion and advantageous inequality aversion). For ease of understanding, we copied the two menus in Tables 3A,B. Each menu consists of 10 choices where the participant has to select between option A and option B. For a rational participant, he/she will keep choosing option A until he/she jumps to option B at a certain line and continues to choose option B in the remaining choices (of course, it is also possible to choose option A or option B in all lines). We call the point at which the participant jumps from option A to option B as the “jump point.” For instance, if the participant chooses option A from the first to the third choices and chooses option B from the fourth to last choices, then his/her jump point is 3. The jump point is set at 10 if the participant always chooses option A and is set at 0 if the participant always chooses option B. According to Yang et al. (2016), we can calculate the range of the envy parameter α and the guilt parameter β of the participant according to his/her jump point, which is also displayed in Tables 3A,B.

**TABLE 3A:** Disadvantageous inequality choice menu.

**No.**	**Option A**	**Option B**	**Choose B if:**
1	Yours: 125; Others: 150	Yours: 100; Others: 260	α ≤ −0.19
2	Yours: 115; Others: 150	Yours: 100; Others: 260	α ≤ −0.12
3	Yours: 105; Others: 150	Yours: 100; Others: 260	α ≤ −0.04
4	Yours: 95; Others: 150	Yours: 100; Others: 260	α ≤ 0.05
5	Yours: 85; Others: 150	Yours: 100; Others: 260	α ≤ 0.16
6	Yours: 75; Others: 150	Yours: 100; Others: 260	α ≤ 0.29
7	Yours: 65; Others: 150	Yours: 100; Others: 260	α ≤ 0.47
8	Yours: 55; Others: 150	Yours: 100; Others: 260	α ≤ 0.69
9	Yours: 45; Others: 150	Yours: 100; Others: 260	α ≤ 1.00
10	Yours: 35; Others: 150	Yours: 100; Others: 260	α ≤ 1.44

**TABLE 3B:** Advantageous inequality choice menu.

**No.**	**Option A**	**Option B**	**Choose B if:**
1	Yours: 185; Others: 90	Yours: 170; Others: 50	β ≤ −0.60
2	Yours: 175; Others: 90	Yours: 170; Others: 50	β ≤ −0.14
3	Yours: 165; Others: 90	Yours: 170; Others: 50	β ≤ 0.11
4	Yours: 155; Others: 90	Yours: 170; Others: 50	β ≤ 0.27
5	Yours: 145; Others: 90	Yours: 170; Others: 50	β ≤ 0.38
6	Yours: 135; Others: 90	Yours: 170; Others: 50	β ≤ 0.47
7	Yours: 125; Others: 90	Yours: 170; Others: 50	β ≤ 0.53
8	Yours: 115; Others: 90	Yours: 170; Others: 50	β ≤ 0.58
9	Yours: 105; Others: 90	Yours: 170; Others: 50	β ≤ 0.62
10	Yours: 95; Others: 90	Yours: 170; Others: 50	β ≤ 0.65

All the participants in a session will be randomly sorted in a circulation, and each one has a “previous player” and a “next player.” For example, player 2 is the previous player of player 3 and the next player of player 1; player 1 is the previous player of player 2 and the next player of the last player. When the participants finish these choices, the computer will randomly select one choice from the 20 choices. The participant’s payoff consists of two parts: the money he/she distributed to himself/herself and the money his/her previous player distributed to him/her. For instance, if the computer selects the third choice of the first menu, and the jump point of player 1, player 2, and player 3 is 0, 5, and 2, respectively, then player 2 will get 365 tokens (105 + 260) and player 3 will get 250 tokens (100 + 150).

### Procedure

The experiment contains two tasks. In the first task, the participants were asked to do the 3-player prisoner’s dilemma for 12 rounds. In each round, the participants were regrouped using the random stranger method. For the 12 rounds, 6 rounds applied SPP and 6 rounds applied TPP, but the order was randomized. After the 12 rounds, the computer will randomly choose one round from the 12 rounds and the total profit of the task is decided by the profit in the chosen round. The exchange rate of tokens and RMB yuan in the first task is 1:1. In the second task, the participants were asked to finish the two inequality choice menus. The exchange rate of tokens and RMB yuan in the second task is 10:1. Afterward, the participants would see their summed profit of the two tasks, i.e., they did not know what they got from each of the tasks and thus could not infer how much punishment they received. However, if the computer chose the revealed condition, the participant would also be told how they performed in the chosen round in the first task and how much punishment they received.

The experiment started with 20 min of tDCS, during which the participants were asked to rest in their chairs with the tDCS devices on their heads. After that the devices were moved away to avoid any discomfort. Then, the experimenters explained the instructions of task 1 and the participants had to go through several testing questions before entering task 1 to make sure that they understood the task. After the first task, the experimenters explained the instructions of task 2, and the participants also needed to pass some testing questions. After the participants completed task 2, they were able to see their total profit from the tasks. All participants were also asked to complete a questionnaire concerning personal information and experiment-related feelings before receiving their payments. The payments of the participants were the combination of a show-up fee of 20 RMB yuan and the profit in the experiment. All tasks were written by the experimental software z-Tree (Fischbacher, 2007).

## Results

### Cooperation Behavior

We will first discuss the participants’ cooperation behavior. The overall cooperation rate is 31.57% in the unilateral treatment (UT) and 37.96% in the bilateral treatment (BT). For UT, 63 of the 90 participants cooperated at least once in the 12 rounds, and the other 27 participants (30%) chose to defect in all rounds. For BT, 16 of the 72 participants (22.22%) chose to defect in all rounds. In both treatments, the cooperation rate gradually decreased from 34.44% (UT) and 51.39% (BT) in the first round to 27.78% (UT) and 33.33% (BT) in the last round. However, the probability of participants choosing to cooperate in the last round was still significantly positive (*t*-test; UT: *p* < 0.001, BT: *p* < 0.001). This finding is consistent with the phenomenon that the cooperation rate decreases over rounds as well as the end-game effect (the cooperation rate in the last round reaches the minimum) universally observed in experiments involving cooperation (Isaac et al., 1982, 1984; Banks et al., 1988).

**FIGURE 2:**
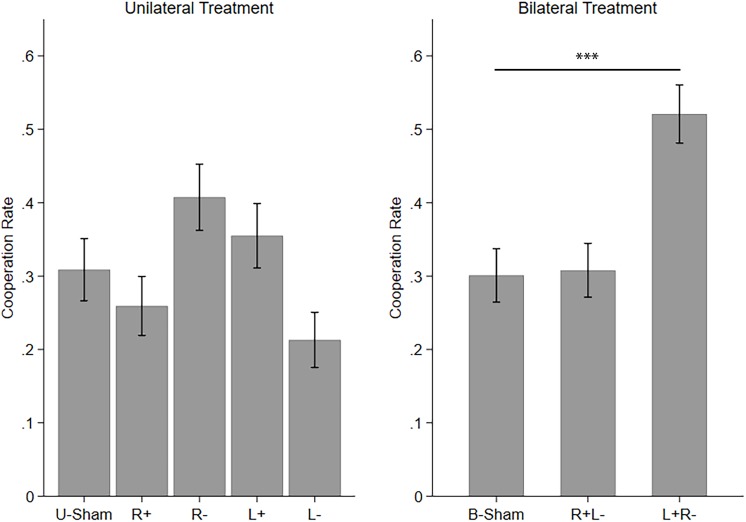
Mean cooperation rates in UT and BT. Error bars indicate 95% confidence intervals. Asterisks indicate statistically significant differences.

Then, we applied one-way ANOVAs and *post hoc* comparisons with Bonferroni correction to test the difference among the stimulation modes in UT and BT. We found that the five stimulation modes in UT are significantly different (*F*_(__4_,_1075__)_ = 6.694, *p* < 0.001, partial η^2^ = 0.024, power = 0.993). However, none of the four active stimulation modes had significant differences compared to U-Sham. The significance of the ANOVA resulted from the differences among the four active stimulation modes themselves. The three stimulation modes in BT are also significantly different (*F*_(__2_,_861__)_ = 21.040, *p* < 0.001, partial η^2^ = 0.047, power = 1.000), with left anodal/right cathodal stimulation significantly increasing cooperation (*p* < 0.001) compared to B-Sham. Figure 2 demonstrates the mean cooperation rates of each stimulation mode in UT and BT.

### Punishment Behavior

#### Data Overview

According to the cooperation behaviors of the imposer and the receiver of the punishment, we can categorize punishment into four types (we denote this as CD-type): from cooperator toward defector (C–D), from cooperator toward cooperator (C–C), from defector toward defector (D–D), and from defector toward cooperator (D–C). This categorization is very important because an experimental study has shown that the punishment patterns of cooperators and defectors are highly different, and the behavior of the punished person plays the most important role in the punishment (Falk et al., 2005). In other words, these four CD types represent different behavioral incentives and thus should not be mixed up. In addition, it is also suggested by previous literature that the SPP and the TPP (we denote this as puni-type) are very different both behaviorally and neurally (Corradi-Dell’Acqua et al., 2012; Leibbrandt and López-Pérez, 2012; Sellaro et al., 2016). Therefore, we have four CD types and two puni types, which leads to an overall 4 × 2 = 8 types of punishment.

**FIGURE 3:**
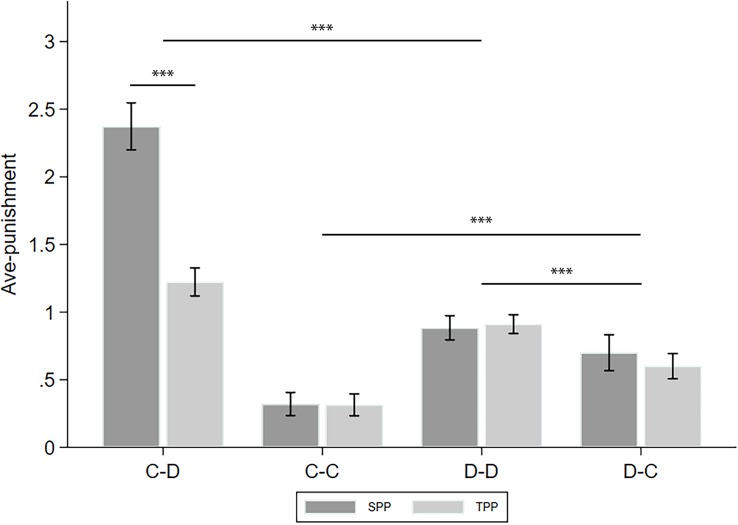
Ave-punishment of the four CD types in the SPP and TPP rounds. Error bars indicate 95% confidence intervals. Asterisks indicate statistically significant differences.

To simplify, we took the average of the punishment in the revealed condition and that in the naive condition and denote this as ave-punishment. The analyses of the differences between the punishments in the revealed and naive conditions (denoted as info-type) are left to the following subsections. Figure 3 displays the ave-punishment of the four CD types in the SPP and TPP rounds. We applied two-way ANOVA with *puni-type* and *CD-type* as the factors to see how they affect ave-punishment. Moreover, we calculated the number of cooperators in the group of the receiver of the punishment (denoted as *C-num*) as a control variable. We found a significant effect of *puni-type* (*F*_(__1_,_4851__)_ = 35.788, *p* < 0.001, partial η^2^ = 0.007, power = 1.000), *CD-type* (*F*_(__3_,_4851__)_ = 149.593, *p* < 0.001, partial η^2^ = 0.085, power = 1.000), and their interaction *puni-type*^∗^*CD-type* (*F*_(__3_,_4851__)_ = 36.911, *p* < 0.001, partial η^2^ = 0.022, power = 1.000). *Post hoc* analyses with Bonferroni correction showed that C–D punishment was significantly higher than D–D punishment (*p* < 0.001), and the latter was significantly higher than D–C punishment (*p* < 0.001). Finally, D–C punishment was significantly higher than C–C punishment (*p* < 0.001). These observations verified that the punishment patterns of cooperators and defectors are highly different. For example, Falk et al. (2005) believed that the defectors’ punishments were mainly based on spitefulness, while the cooperators’ punishments were mainly based on reciprocity. In addition, we found that the SPP is significantly higher than the TPP only in C–D punishment (*p* < 0.001). Since the SPP is usually regarded as a combination of personal (or direct) reciprocity and norm maintenance while the TPP only includes the latter (Bowles and Gintis, 2004; Falk and Fischbacher, 2006), this difference measures the portion of personal reciprocity of the cooperators toward the defectors. On the other hand, the defectors may not have a strong intention of personal reciprocity when issuing punishments.

#### Second-Party Punishment

**FIGURE 4:**
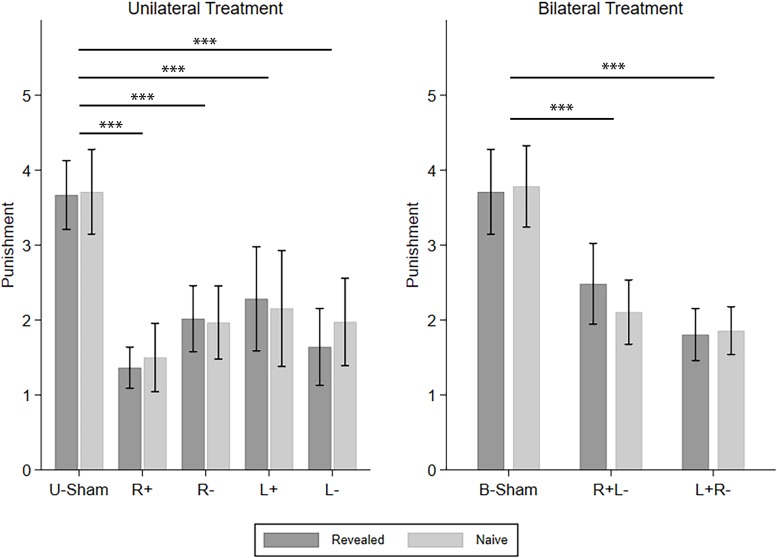
Means of the SPP in the revealed and naive conditions under different tDCS modes. Error bars indicate 95% confidence intervals. Asterisks indicate statistically significant differences.

Then, we investigated how different types of SPP reacted to different tDCS modes, i.e., the five UT modes and the three BT modes. The four CD types of punishment were investigated one by one using repeated-measures ANOVA with *info-type* as the within-subject variable, *stim* (R+/R−/L+/L−/U-Sham for UT, R+L−/L+R−/B-Sham for BT) as the between-subject variable, and *C-num* as the control variable. For C–D punishment in UT, we found a significant effect of *stim* (*F*_(__4_,_224__)_ = 9.303, *p* < 0.001, partial η^2^ = 0.142, power = 1.000). *Post hoc* analyses with Bonferroni correction showed that all four active stimulation modes in UT significantly decreased the punishment compared to U-Sham (R+: *p* < 0.001, R−: *p* < 0.001, L+: *p* < 0.001, L−: *p* < 0.001). For C–D punishment in BT, we also found a significant effect of *stim* (*F*_(__2_,_186__)_ = 15.226, *p* < 0.001, partial η^2^ = 0.141, power = 0.999) and further demonstrated that both right anodal/left cathodal and left anodal/right cathodal stimulations significantly decreased the punishment compared to B-Sham (R+L−: *p* < 0.001, L+R−: *p* < 0.001) in *post hoc* analysis with Bonferroni correction. For C–C punishment, we did not find any significant effect in either UT or BT. For D–D punishment in UT, we found a significant effect of *stim* (*F*_(__4_,_480__)_ = 4.763, *p* = 0.001, partial η^2^ = 0.038, power = 0.953), but none of the four active stimulation modes had significant differences compared to U-Sham; for D–D punishment in BT, no significant effect was found. For D–C punishment in UT, *stim* also had a significant effect (*F*_(__4_,_224__)_ = 2.862, *p* = 0.024, partial η^2^ = 0.049, power = 0.777), but none of the four active stimulation modes had significant differences compared to U-Sham. However, for D–C punishment in BT, there was a significant effect of *stim* (*F*_(__2_,_186__)_ = 3.192, *p* = 0.043, partial η^2^ = 0.033, power = 0.605) and the interaction of *stim* and *info* (*F*_(__2_,_186__)_ = 4.044, *p* = 0.019, partial η^2^ = 0.042, power = 0.721). *Post hoc* analyses with Bonferroni correction showed that right anodal/left cathodal stimulation significantly increased the punishment in the naive condition compared to B-Sham (*p* = 0.013). As our main findings focus on C–D punishment, Figure 4 demonstrates the means of the C–D punishments under different stimulation modes and different info-types as an overview of how different stimulations modify SPP.

#### Third-Party Punishment

**FIGURE 5:**
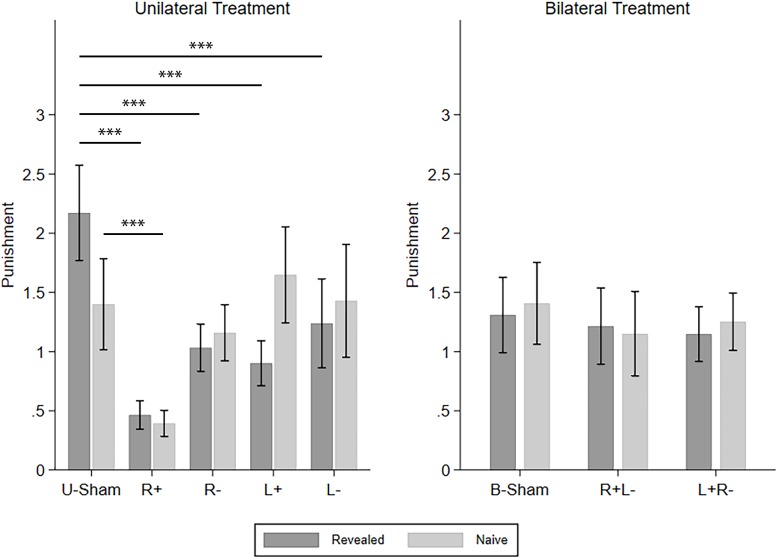
Means of the TPP in the revealed and naive conditions under different tDCS modes. Error bars indicate 95% confidence intervals. Asterisks indicate statistically significant differences.

Similarly, we continued to investigate how different types of TPP reacted to the different tDCS modes. The four CD types of punishment were investigated one by one using repeated-measures ANOVA with *info-type* as the within-subject variable, *stim* (R+/R−/L+/L−/U-Sham for UT, R+L−/L+R−/B-Sham for BT) as the between-subject variable, and *C-num* as the control variable. For C–D punishment in UT, we found a significant effect of *stim* (*F*_(__4_,_296__)_ = 9.253, *p* < 0.001, partial η^2^ = 0.111, power = 1.000) and the interaction of *stim* and *info* (*F*_(__4_,_296__)_ = 9.381, *p* < 0.001, partial η^2^ = 0.113, power = 1.000). *Post hoc* analyses with Bonferroni correction showed that all four active stimulation modes in UT significantly decreased the punishment compared to U-Sham in the revealed condition (R+: *p* < 0.001, R−: *p* < 0.001, L+: *p* < 0.001, L−: *p* = 0.003), while only right anodal stimulation significantly decreased the punishment compared to U-Sham in the naive condition (*p* = 0.006). On the other hand, for C–D punishment in BT, no significant effects were found. For C–C punishment, again, no significant effects were found. For D–D punishment in UT, we found a significant effect of *stim* (*F*_(__4_,_835__)_ = 7.416, *p* < 0.001, partial η^2^ = 0.034, power = 0.996) and *info-type* (*F*_(__1_,_835__)_ = 10.148, *p* = 0.001, partial η^2^ = 0.012, power = 0.889). *Post hoc* analyses with Bonferroni correction showed that right anodal stimulation significantly decreased the punishment compared to U-Sham (*p* = 0.002) and that the punishment in the naive condition was significantly higher than that in the revealed condition (*p* < 0.001). For D–D punishment in BT, a significant effect of *stim* was also found (*F*_(__2_,_523__)_ = 3.222, *p* = 0.041, partial η^2^ = 0.012, power = 0.608), but none of the active stimulation modes had significant differences compared to B-Sham. For D–C punishment in UT, we also found a significant effect of *stim* (*F*_(__4_,_296__)_ = 10.842, *p* < 0.001, partial η^2^ = 0.128, power = 1.000) and *info-type* (*F*_(__1_,_296__)_ = 5.816, *p* = 0.016, partial η^2^ = 0.019, power = 0.665). *Post hoc* analyses with Bonferroni correction showed that left cathodal stimulation significantly increased the punishment compared to U-Sham (*p* = 0.001) and that the punishment in the naive condition was significantly higher than in the revealed condition (*p* = 0.003). Finally, for D–C punishment in BT, the effect of *stim* was also significant (*F*_(__2_,_285__)_ = 3.060, *p* = 0.048, partial η^2^ = 0.021, power = 0.589), but none of the active stimulation modes had significant differences compared to B-Sham. Again, as our main findings focus on C–D punishment, Figure 5 demonstrates the means of the C–D punishments under different stimulation modes and different info-types as an overview of how different stimulations modify TPP.

### Inequality Aversion

Finally, we examined the participants’ levels of inequality aversion. When introducing the inequality choice menus, we mentioned that we can calculate the range of the envy parameter α and the guilt parameter β of the participant according to his/her jump point. We used the median of the range interval as an approximation of the parameter. For example, if the participant’s jump point was 4 in the disadvantageous inequality choice menu, then his/her envy parameter α is located within the interval (0.05, 0.16]; therefore, we took (0.05 + 0.16)/2 = 0.105 as the approximation of α. If the participant’s jump point was 10 or 0, we used the maximum or minimum value of the possible α, i.e., −0.19 or 1.44. The same approach was also employed for the guilt parameter β. There were four participants who did not have jump points in both the disadvantageous inequality choice menu and the advantageous inequality choice menu because they jumped many times between options A and B and thus were obviously irrational. Another participant displayed irrationality only in the advantageous inequality choice menu. Therefore, we treated these data as missing values. One-way ANOVA tests showed that there was no significant difference among the UT stimulation modes or the BT stimulation modes for α (UT: *F*_(__4_,_87__)_ = 0.387, *p* = 0.817, partial η^2^ = 0.018, power = 0.139; BT: *F*_(__2_,_69__)_ = 0.129, *p* = 0.879, partial η^2^ = 0.004, power = 0.063) and β (UT: *F*_(__4_,_87__)_ = 0.459, *p* = 0.765, partial η^2^ = 0.022, power = 0.217; BT: *F*_(__2_,_68__)_ = 1.408, *p* = 0.252, partial η^2^ = 0.041, power = 0.304). This means that the stimulation did not change the participant’s preferences for inequality.

### Summary

As we have many stimulation modes and different types of behavior, we would like to summarize our findings in this subsection for a clearer understanding of how modulating the activity in the DLPFC affects the participant’s behaviors in our study. We found that the left anodal/right cathodal stimulation significantly increased cooperation compared to B-Sham. For SPP, we found that the right anodal, right cathodal, left anodal, and left cathodal stimulations significantly decreased the C–D punishment compared to U-Sham, and both the right anodal/left cathodal and left anodal/right cathodal stimulations significantly decreased the C–D punishment compared to B-Sham. In addition, the right anodal/left cathodal stimulation significantly increased the D–C punishment in the naive condition compared to B-Sham. For TPP, the right anodal, right cathodal, left anodal, and left cathodal stimulations significantly decreased the C–D punishment compared to U-Sham in the revealed condition, while only the right anodal significantly decreased the C–D punishment compared to U-Sham in the naive condition. In addition, the right anodal stimulation significantly decreased the D–D punishment compared to U-Sham, and the left cathodal stimulation significantly increased the D–C punishment compared to U-Sham. However, none of the stimulations changed the participant’s preferences for inequality. Table 4 summarizes these findings.

**TABLE 4:** Summary of the effect of tDCS on behavior.

		**Unilateral treatment**				**Bilateral treatment**	
		**R+**	**R−**	**L+**	**L−**	**R+L−**	**L+R−**
**Cooperation**							↑
2*SPP	C–D	↓	↓	↓	↓	↓	↓
	D–C					↑(N)	
3*TPP	C–D	↓	↓(R)	↓(R)	↓(R)		
	D–D	↓					
	D–C				↑		

*N in parenthesis means only in the naive condition and R in parenthesis means only in the revealed condition.*

## Discussion

Altruistic punishment of social norm violations plays a crucial role in maintaining widespread cooperation in human societies, and punitive behavior has been suggested to be related to the activity level of the DLPFC. This study used unilateral and bilateral tDCS to investigate how modulating the activity of the DLPFC corresponds to cooperation and punishment in a 3-player prisoner’s dilemma. We found that none of the unilateral stimulations changed the participants’ cooperation behavior, while the left anodal/right cathodal stimulation increased the participants’ cooperation. For punitive behavior, we found that all unilateral stimulations (i.e., right anodal, right cathodal, left anodal, left cathodal) and bilateral stimulations (i.e., right anodal/left cathodal, left anodal/right cathodal) significantly decreased the participant’s SPP imposed by the cooperators toward the defectors. In addition, right anodal stimulation significantly decreased the participant’s TPP imposed by the cooperators toward the defectors. The other three unilateral stimulations also significantly decreased the participant’s TPP imposed by the cooperators toward the defectors, but only when the punishment was revealed to the punished person. Finally, right anodal/left cathodal stimulation increased the punishment imposed by defectors toward cooperators when punishment was not revealed to the punished person; right anodal stimulation decreased the punishment imposed by the defectors toward the defectors; and left cathodal stimulation increased the punishment imposed by the defectors toward the cooperators.

The contribution of our study lies in the following aspects. First, this is the first study to investigate how modulating the activity in the DLPFC affects punishment behaviors in a 3-player prisoner’s dilemma. Previous neural studies have tended to use the ultimatum game to frame punishment behavior because it is simple to implement. In the ultimatum game, rejecting an offer is regarded as punishment (Henrich et al., 2001; Camerer, 2003a, b; Fehr and Fischbacher, 2004). However, this kind of punishment is quite different from what we mean by punishing norm violations in cooperation, and it is important to check whether the DLPFC has the same functioning mechanism in the ultimatum game and in prisoner’s dilemma. Second, we investigated the effect of four unilateral and two bilateral tDCS modes on punishment behavior, exploring how the effect of changing the hemispherical balance of the activity levels of the right and left DLPFC differs from the combinational effect of separately changing the activity levels of the right and left DLPFC. Third, this study is the first to compare the stimulation effects on SPP and TPP, which are suggested by experimental studies to have different behavioral modes (Fehr and Fischbacher, 2004; Buckholtz et al., 2008). Fourth, we disentangled the four different types of punishment according to the cooperation behaviors of the imposer and the receiver of the punishment. Previous neural studies have not paid attention to the heterogeneity of the participants with respect to cooperation tendency, while behavioral studies have shown that cooperators and defectors have different logics in punishing others and that there are also punishments toward the cooperators, i.e., antisocial punishments (Nikiforakis, 2008; Rand et al., 2010; Hauser et al., 2014). Last, we tested whether the stimulation effect is different when the receiver of the punishment was informed about the punishment or not because the two conditions may involve different incentives. We also tested whether the stimulations had changed the participants’ levels of inequality aversion, which is a very important consideration when making punitive decisions (Masclet and Marie-Claire, 2008; Raihani and McAuliffe, 2012; Thöni, 2014).

Our results showed that modulating the activity in the DLPFC has notably different effects on the punishment behavior toward norm defection compared to that in the ultimatum game, which implies that we should be very careful when extending the conclusions about one kind of punishment to another. We also found that the effect of changing the hemispherical balance of the activity level of the right and left DLPFC may not be equivalent to the simple combinational effect of changing separately the activity level of the right and left DLPFC, especially with regard to TPP (Sellaro et al., 2016). This indicates that the two hemispheres of the DLPFC may be systematically connected when processing the punitive decision, and it is important to consider this when exploring the neural basis of punishment behavior. In addition, we found that the effects of stimulations on the cooperators’ punishments and the defectors’ punishments are largely different. For instance, left cathodal stimulation and right anodal/left cathodal stimulation decreased the former and enhanced the latter. Experimental evidence has shown that the cooperators’ punishments are mainly based on reciprocity, while defectors’ punishments are mainly based on spitefulness (Fehr and Gächter, 2002; Carpenter and Matthews, 2004; Rockenbach and Milinski, 2006; Rand et al., 2010; Hauser et al., 2014). One possibility could be that the stimulations decreased the subject’s sense of justice, making the cooperators less willing to maintain justice – i.e., punish the defectors – and the defectors less guilty about their spitefulness. In addition, there was a slight difference between the stimulation effects on SPP and TPP with regard to whether the punishment was revealed to the punished person. This may be because people focus more on teaching the defectors a lesson rather than harming the defectors in the TPP compared to the SPP, and it is the willingness to teach the defectors a lesson that is more sensitive to the stimulations. Last, we found that the stimulation effects were not due to the change in the participants’ levels of inequality aversion. This result is consistent with Ruff et al. (2013), who found that right lateral prefrontal cortex stimulation did not affect awareness of the fairness norm and expected sanctions. Previous studies have suggested that the putamen encodes efficiency, whereas the insula represents inequity and the caudate/septal subgenual area responds to a trade-off between efficiency and inequity (Hsu et al., 2008). This may indicate that the stimulations in our study did not affect the information processing in these parts.

Most importantly, we found that all unilateral stimulations (i.e., right anodal, right cathodal, left anodal, left cathodal) and bilateral stimulations (i.e., right anodal/left cathodal, left anodal/right cathodal) significantly decreased the participant’s SPP imposed by the cooperators toward the defectors. Previous studies have indicated two possible mechanisms through which the DLPFC could modulate the decision-making of altruistic punishment. One is the mechanism of selfishness (Knoch et al., 2006, 2007, 2010; Baumgartner et al., 2011; Buckholtz et al., 2015). This means that the activation of the DLPFC enables people to better control their selfishness, thus leading to more selfless behaviors. Therefore, people will be more willing to punish norm violators at their own expense if they have a higher level of activation in the DLPFC and will be less willing to punish violators if they have a lower level of activation in the DLPFC. The other is the mechanism of negative emotions (Sanfey et al., 2003; Wu and Zhou, 2012; Luo et al., 2013; Yin et al., 2019). This means that the activation of the DLPFC enables people to better control their negative emotions toward the norm violators, thus making them not as willing to punish those norm violators. As a result, people will impose lower altruistic punishments if they have a higher level of activation in the DLPFC and will impose higher altruistic punishments if they have a lower level of activation in the DLPFC. Recalling our hypotheses, our findings indicate that the mechanisms of selfishness and negative emotions most likely interact with different stimulations. More specifically, for anodal stimulations, the mechanism of negative emotions may overwhelm the mechanism of selfishness, while for cathodal stimulations, the mechanism of selfishness may be more dominant than the mechanism of negative emotions. This implies that the neural basis of the DLPFC in processing punitive decisions may be more complicated than previously believed. This finding could indicate, for example, that modulating the activity in the DLPFC, whether with enhancement or inhibition, may evoke a series of reactions that activate the two mechanism to different levels. In fact, the situation is likely as described by Buckholtz and Marois (2012), who stated that the anatomical connectivity (Croxson et al., 2005) and context-dependent functions of the prefrontal cortex (Duncan, 2010) make it more likely that the stimulated lateral prefrontal cortex area integrates and coordinates activity in a network of brain regions triggered by the need for considering social punishments during action control.

## Data Availability Statement

The datasets generated for this study are available on request to the corresponding author.

## Ethics Statement

The studies involving human participants were reviewed and approved by the Zhejiang University Ethics Committee. The patients/participants provided their written informed consent to participate in this study.

## Author Contributions

SC, JS, XY, HY, and JL designed the experiment, wrote and revised the manuscript, and finally approved the version to be published. SC and JL performed the experiment and analyzed the data. SC drew the figures.

## Conflict of Interest

The authors declare that the research was conducted in the absence of any commercial or financial relationships that could be construed as a potential conflict of interest.

## Footnotes

1 The ages of the participants range from 17 to 27, except one who is 31 years old.
